# The Search for Covalently Ligandable Proteins in Biological Systems

**DOI:** 10.3390/molecules21091170

**Published:** 2016-09-02

**Authors:** Syed Lal Badshah, Yahia Nasser Mabkhot

**Affiliations:** 1 Department of Chemistry, Islamia College University Peshawar, Peshawar 25120, Pakistan; 2 Department of Biochemistry, Abdul Wali Khan University, Mardan, Khyber Pukhtoonkhwa 25120, Pakistan; 3 Department of Chemistry, College of Science, King Saud University, P.O. Box 2455, Riyadh 11451, Saudi Arabia; yahia@ksu.edu.sa

**Keywords:** fragment-based ligand discovery, covalent binding ligands, druggable cysteines, isotopic tandem orthogonal proteolysis‒activity-based protein profiling, methyl transferase, protease caspase enzyme

## Abstract

This commentary highlights the recent article published in Nature, June 2016, titled: “Proteome-wide covalent ligand discovery in native biological systems”. They screened the whole proteome of different human cell lines and cell lysates. Around 700 druggable cysteines in the whole proteome were found to bind the electrophilic fragments in both active and inactive states of the proteins. Their experiment and computational docking results agreed with one another. The usefulness of this study in terms of bringing a change in medicinal chemistry is highlighted here.

The role of medicinal compounds that bound in covalent fashion such as penicillin, aspirin, and proton pump inhibitors are still playing their part in benefiting humankind [1]. Covalent inhibitors development is important due to drug resistance and inefficiency of many cancerous drugs [1]. Recently, a number of oncogenic proteins have been targeted by covalent inhibitors and these include KRAS [2]; mutated kinases [3]; and other kinases in general [4,5,6,7,8]. It is also a common view that the chances of resistance to covalent inhibitors is low [9]. They are used in very small amounts and have high specificity, but the limitation is that of its toxicity. The side effects of covalent drug inhibitors can be controlled through selective measures such as better designing and considering proper electrophilic properties of the medicinal compounds [1]. There are approximately 40 drugs available in the market that covalently binds to its target protein [1]. Some of these drugs are in clinical trials, but big pharmaceutical companies are still reluctant to invest and research on these pharmaceuticals due to their side effects [1]. The covalent ligands sometimes bind very tightly with plasma proteins and cells and result in allergic reaction and tissue damage [1].

Previously, the widespread human proteome kinases have been targeted by covalent inhibitors [10], and medicinal chemists have also been working on targeting the overall proteome. Backus et al. (2016) of the Scripps Research Institute, CA, USA, published an article in Nature in which they quantitatively checked parts of small molecules that are reactive to cysteines of the thousands of proteins from the human proteome and cells [11]. They took on the daunting challenge of searching for covalent ligands using the fragment-based ligand discovery (FBLD) method. The major difficulty of this approach is that it needs a purified protein, so that the drug can bind in its specific pocket and its action can be observed. This limitation hampers the efforts to study all of the functional proteins through this method, as it is not possible to purify so many proteins [11]. However, the fragment-based ligand discovery is now almost an established technique. It is used by big pharmaceutical companies such as AstraZeneca [12] for a number of challenging proteins that are important from a medicinal point of view. Better fitting and proper binding can enhance the efficiency of FBLD and this is one of its advantages. Three things are essential in covalent-based inhibitors research; these are cysteines in the protein, mass spectrometer, and the electrophile library compounds.

In this study, Backus et al. identified more than 700 cysteine residues in the whole proteome that can bind covalent ligands [11]. In the past, the cysteine-captured ligand strategy of proteins was exploited by Erlanson and coworkers, where they used a lower concentration (10–200 μM) of the covalent binding drugs [13]. In such a strategy, the protein-ligand complex was quite stable and was also detected by mass spectrometry [13]. However, Backus et al. used a ligand concentration of 500 μM in this work, which is more than double that of Erlanson et al. and is quite expensive in terms of cost.

A covalent ligand-protein docking protocol called DOCKTITE is available in the Molecular Operating Environment (MOE) software along with virtual screening [14]. It is also significantly advantageous to the community of medicinal chemists that they can model most of the proteins of human proteome from the UNIPROT web server and tested them for thousands of electrophilic organic compounds available in various online libraries [15,16,17,18,19,20]. Backus et al. also used the computational docking approach to find the covalent ligandable cysteines in the proteome using the Autodock software, and their computation results agreed well with that of experimental ones from isoTOP-ABPP [11].

They functionally tested their method of isoTOP-ABPP by assaying two proteins named methyl transferase and MAP 3 kinase, which contain ligandable cysteines residues, with proved activities. These two enzymes gave positive results in terms of inhibition by the selected drugs [11]. Then, they evaluated the activity of nucleotide biosynthetic enzyme IMPDH2 and p53-induced phosphatase TIGAR by their method, and these two proteins functions were affected when ligands bound with their cysteine, showing the success of the isotope-ABPP method [11]. They also tested several other enzymes involved in cancer such as isocitrate dehydrogenases 1 and 2, which contain conserved cysteines in vitro and in vivo, and found suitable ligands for them. They tested the different protease caspase enzymes in various human cell lines and obtained mixed results [11]. Through the fragment-based covalent ligand approach, the whole proteome of a subject can be screened and gives information about all proteins that can bind covalent ligands in active and inactive forms during their various biological functions [11]. These druggable cysteines in the active and inactive states of proteins showed specific structure–activity relationships (SARs) with the available fragment electrophile compounds that can be modified in a more efficient way with less toxicity [11].

Although working on a large scale with a proteome screen, a great number of proteins and small covalent molecules will be specified, but there are a number of disadvantages. For example, one covalent inhibitor will fit in more than one different protein pocket and will inhibit the activity of several metabolic pathways at a time. Thus, toxicity will be the main hindrance in studying several proteins at a time. Therefore, a strategy should be designed where only one covalent inhibitor targets a specific protein with high selectivity, and in that way we can lower the toxicity.

The availability of the human genome, protein modeling techniques, and the FBLD approach is an opportunity that should be exploited by academia and industry for covalent-based drug research, so that a cure for a number of diseases can be sorted out.
